# Supplementary material for: Identification and Genome Characterization of a Novel Virus within the Genus Totivirus from Chinese Bayberry (Myrica rubra)
Source: Viruses. 2024 Feb 12;16(2):283. doi: 10.3390/v16020283 (PMC10893191; doi:10.3390/v16020283)
Supplement: Supplementary file 1 [file viruses-16-00283-s001.zip › Supplementary Table 1.Primers.pdf]

Supplementary Table 1 Primers used for RT-PCR amplification of MRaTV1

Primer	Sequence (5' to 3')	Position[1]	Amplicon size (bp)
MRaTV1-5'GSP1	GTCCATATCTAGTGTCAGGTGC	399-421	421
MRaTV1-5'GSP2	GCATCTTAGCAACGTGCGCTGA	253-275	275
(L1) MRaTV1-F1	GACGACAAGCCAAACACCACAAC	1-23	1,075
(L1) MRaTV1-R1	CTGTGCGAATACGAGTGCCATCAG	1,051-1,075	
(L2) MRaTV1-F2	GAAGCAACCTACTGGCTATCAG	889-911	986
(L2) MRaTV1-R2	CGTCAGTTTCATTAGTATGGGGC	1,851-1,874	
(L3) MRaTV1-F3	GATGGTTGATGACAAGATAGGCAAG	1,674-1,699	1004
(L3) MRaTV1-R3	CTGTCATCTCTCCGTAGTCATAGC	2,653-2,677	
(L4) MRaTV1-F4	CAGGACGGGAGTGTCAGGTTG	2,480-2,500	902
(L4) MRaTV1-R4	CACGAACTTGGTCCTGTGCCTGGCTGT	3,359-3,381	
(L5) MRaTV1-F5	ACAGGTTGACCACGAAAG	3,175-3,193	994
(L5) MRaTV1-R5	CTACTCTCGAGTGGGTCATCG	4,147-4,168	
(L6) MRaTV1-F6	CGGTGATGATGTATTACTAGCTGTG	3,953-3,978	753
(L6) MRaTV1-R6	TCCACATACGGCACACTGATC	4,684-4,705	

1. Refer to the position of each primer pair on the genome of the MRaTV1.
